# Prenatal Alcohol Exposure as a Case of Involuntary Early Onset of Alcohol Use: Consequences and Proposed Mechanisms From Animal Studies

**DOI:** 10.3389/fnbeh.2020.00026

**Published:** 2020-03-05

**Authors:** Mirari Gaztañaga, Asier Angulo-Alcalde, M. Gabriela Chotro

**Affiliations:** Departamento de Procesos Psicológicos Básicos y su Desarrollo, Facultad de Psicología, University of the Basque Country UPV/EHU—Donostia-San Sebastián, San Sebastian, Spain

**Keywords:** prenatal, alcohol, learning, opioids, acetaldehyde, associative, reinforcer

## Abstract

Prenatal alcohol exposure has been found to be an important factor determining later consumption of this drug. In humans, despite the considerable diversity of variables that might influence alcohol consumption, longitudinal studies show that maternal alcohol intake during gestation is one of the best predictors of later alcohol use from adolescence to young adulthood. Experimental studies with animals also provide abundant evidence of the effects of prenatal alcohol exposure on later alcohol intake. In addition to increased consumption, other effects include enhanced palatability and attractiveness of alcohol flavor as well as sensitization to its sensory and reinforcing effects. Most of these outcomes have been obtained after exposing rats to binge-like administrations of moderate alcohol doses during the last gestational period when the fetus is already capable of detecting flavors in the amniotic fluid and learning associations with aversive or appetitive consequences. On this basis, it has been proposed that one of the mechanisms underlying the increased acceptance of alcohol after its prenatal exposure is the acquisition (by the fetus) of appetitive learning *via* an association between the sensory properties of alcohol and its reinforcing pharmacological effects. It also appears that this prenatal appetitive learning is mediated by the activation of the opioid system, with fetal brain acetaldehyde playing an important role, possibly as the main chemical responsible for its activation. Here, we review and analyze together the results of all animal studies testing these hypotheses through experimental manipulation of the behavioral and neurochemical elements of the assumed prenatal association. Understanding the mechanisms by which prenatal alcohol exposure favors the early initiation of alcohol consumption, along with its role in the causal pathway to alcohol disorders, may allow us to find strategies to mitigate the behavioral effects of this early experience with the drug. We propose that prenatal alcohol exposure is regarded as a case of involuntary early onset of alcohol use when designing prevention policies. This is particularly important, given the notion that the sooner alcohol intake begins, the greater the possibility of a continued history of alcohol consumption that may lead to the development of alcohol use disorders.

## Introduction

Fetal Alcohol Spectrum Disorder (FASD) refers to the range of adverse effects that can occur in the children of women who consume alcohol during pregnancy (Riley and McGee, 2005; Manning and Eugene Hoyme, 2007). At the most severe end of the spectrum is Fetal Alcohol Syndrome (FAS), which may occur with prolonged consumption of relatively high amounts of alcohol (Jones and Smith, 1973; Jones et al., 1973). Prenatal exposure to alcohol may also produce either partial FAS (some of the diagnostic features occur) or may result in Alcohol-Related Birth Defects, or Alcohol-Related Neurodevelopmental Disorders (Stratton et al., 1996; Johnson et al., 2018) Interestingly, whilst the level of alcohol exposure may determine the severity of the observed effects, other cognitive, neuropsychological, and behavioral deficits have consistently been observed (Streissguth, 1986; Astley et al., 2009; Guerri et al., 2009; Kable et al., 2016; Temple et al., 2019).

Although not recognized as a symptom of FASD, there is evidence for an association between prenatal alcohol exposure and alcohol use disorders in offspring. Despite the considerable range of variables that might influence alcohol consumption by individuals, the few existing prospective longitudinal studies analyzing the relation between prenatal alcohol exposure and alcohol use by offspring all agree on one chief outcome: maternal alcohol intake during gestation is one of the best predictors of later alcohol use from adolescence to young-adulthood (Baer et al., 1998, 2003; Griesler and Kandel, 1998; Alati et al., 2006; Streissguth, 2007; Cornelius et al., 2016; Goldschmidt et al., 2019). Concurrently, numerous experimental studies with animals have shown increased alcohol intake in the offspring of dams that had consumed alcohol during gestation. These studies have been extensively reviewed in three previous publications, in which the effects of prenatal alcohol exposure on infantile, adolescent and adult alcohol acceptance and intake were described, analyzing the possible mechanism involved in those effects (Spear and Molina, 2005; Chotro et al., 2007; Abate et al., 2008). In the present review, we continue to explore this issue by analyzing specific research on the subject from the last 15 years, particularly those studies that have used animal models, focusing on the importance of mechanisms related to fetal learning about alcohol.

## Clinical Evidence

Most of the clinical evidence on the association between prenatal alcohol exposure and alcohol use disorders is derived mainly from a few longitudinal studies. The first of these is the “Seattle Prospective Longitudinal Study on Alcohol and Pregnancy,” in which the effects of prenatal alcohol exposure were examined on a cohort of children born in 1974 to mothers selected as representative of the Seattle/King County population in the USA (Streissguth et al., 1981). In this study, among many other variables, alcohol use problems were measured in the offspring at three different ages: 14, 21 and 25 years. When subjects were 14 years old, three sets of data from 439 families were collected: adolescent alcohol use, family history of alcohol problems, and prenatal alcohol exposure history; and using these data they conducted correlational analyses. Prenatal alcohol exposure was found to be a better predictor of adolescent alcohol use than a family history of alcohol problems (Baer et al., 1998). When these same subjects were 21 years old, the families were re-evaluated, and it was reported that prenatal alcohol exposure was still strongly associated with a higher number of symptoms of alcohol dependence in early adulthood. This relationship persisted independently of the effects of a family history of alcohol problems, nicotine exposure, other prenatal exposures, and postnatal environmental factors including parental use of other drugs (Baer et al., 2003). At the age of 25, the association between prenatal alcohol exposure and adult alcohol use problems was still present (Streissguth, 2007). In all these cases the results were obtained after adjusting for maternal demographic characteristics, maternal use of tobacco and other drugs during pregnancy, and maternal and familial alcohol problems after birth. In line with those outcomes, alcohol exposure during gestation was also found to be a key factor for predicting alcohol use disorders in adults who were adopted at birth (Yates et al., 1998). In one study, 197 adoptees were evaluated for the use of alcohol, tobacco, and other drugs. Of those, 21 had received prenatal alcohol exposure and their outcomes were compared with those of 102 control adoptees who had received no alcohol exposure. The results showed that even when controlling for biological parental alcohol abuse or dependence, the prenatal alcohol exposure factor was still the best predictor of alcohol use disorders. This study highlights the relevance of alcohol exposure during prenatal development for alcohol abuse in adulthood, a relationship that appears to exist independently of confounding postnatal environmental variables (Yates et al., 1998).

Another follow-up study with participants from the “Mater–University of Queensland Study of Pregnancy and Its Outcomes” was designed specifically to analyze the association between maternal alcohol exposure and the onset of alcohol disorders. This study was conducted with 2,138 participants from a population-based birth cohort, born in Brisbane, Australia in 1981 (Alati et al., 2006). Mothers and their sons/daughters were followed from pregnancy to the offspring’s early adulthood, and the onset of alcohol disorders was registered from adolescence to 21 years old. The results revealed that *in utero* exposure to three or more drinks containing alcohol was related to alcohol use disorders at the age of 21, increasing its risk by almost three times compared with subjects exposed to either smaller amounts of alcohol or those that had received no exposure. In addition, they reported that the sons and daughters of mothers who had consumed three or more glasses of alcohol during early pregnancy were almost four times more likely to show an early onset of alcohol disorders at the age of 21 than those whose mothers had consumed less than two drinks at any time. This association was robust, even after adjusting for a number of biological and environmental factors (Alati et al., 2006). In another publication, similar results were reported when subjects were 14 years old (Alati et al., 2008).

Another longitudinal prospective study aimed to analyze the relative contribution of familial risk and prenatal exposure to substance use in offspring. This study was carried out with a sample of 209 third-generation offspring of families from the area of Pittsburgh, USA, specifically selected for being at either high or low risk of developing alcohol dependence (O’Brien and Hill, 2014). High-risk families were selected based on the presence of two alcohol-dependent sisters and low-risk families were selected on the basis of having a minimal number of first and second-degree relatives with alcohol dependence. The results of this study showed that prenatal alcohol exposure increased the risk of alcohol use disorders in both high and low-risk participants, although high-risk mothers were more likely to use alcohol and cigarettes during each trimester of pregnancy. In addition, it was reported that among the high-risk offspring, the effects of prenatal exposure were more specific to the particular substance exposed, i.e., prenatal alcohol exposure was associated with alcohol problems in offspring, while cigarette exposure was associated with cigarette use.

Another study analyzed the link between maternal self-reported alcohol consumption during pregnancy and adolescent self-reported drinking in a sample of 185 mothers and their first-borns recruited from the New York State Cohort (Griesler and Kandel, 1998). Maternal drinking was assessed retrospectively, at an average of 3.3 years after delivery, with reports covering a period of 18 months (including pregnancy). Adolescent (age 9–17) life-time and current alcohol drinking data were obtained from self-reports. The results indicated that maternal alcohol drinking during pregnancy—particularly moderate to heavy consumption—was associated with the current drinking of their female offspring. No association was found, however, between maternal drinking, either during or after pregnancy, and alcohol drinking in sons.

Finally, a fifth longitudinal study was conducted with participants from cohorts of the “Maternal Health Practices and Child Development Project.” These participants were recruited between 1983 and 1986, also from Pittsburg, USA, and they have been followed since the fourth gestational month. The data from this study demonstrated that the level of adolescent drinking (at the age of 16) was directly predicted by prenatal alcohol exposure, as well as lower levels of parental strictness and exposure to maltreatment and violence during childhood (Cornelius et al., 2016). These authors also reported that heavier drinking during adolescence is directly predicted by maternal alcohol consumption during pregnancy. A similar relation between these variables was observed when analyzing data collected during young adulthood, at the age of 22 (Goldschmidt et al., 2019). The results of these studies, together with the one described previously, provide clear evidence of the direct connection between maternal and adolescent drinking, which not only includes alcohol dependence and alcohol-related problems.

In sum, the outcomes of all these studies support the existence of an association between prenatal alcohol exposure and either early onset of alcohol drinking and/or with the development of alcohol use disorders in adolescence and young adulthood. In addition, most of these studies highlight a critical role for prenatal alcohol exposure in the idea of a causal pathway that leads to alcohol use disorders. Several mechanisms have been proposed to underlie the link between prenatal alcohol exposure and these consequences, although research studies at this level have primarily been conducted with laboratory animals.

## Evidence From Studies With Animals

Experimental studies with animals provide abundant evidence confirming the results found in humans i.e., prenatal exposure to alcohol—in addition to producing numerous harmful effects—will, in most cases, be followed by an increased acceptance (i.e., attraction and consumption) of alcohol. These effects have been reviewed comprehensively in previous publications in which the outcomes of numerous studies with rodents exposed prenatally and perinatally to alcohol are described and analyzed, focusing on the behavioral effects of prenatal exposure, and in particular, on the factors that play a role in the postnatal response to alcohol (Spear and Molina, 2005; Chotro et al., 2007; Abate et al., 2008). Table 1 includes the studies in rodents described in those reviews, as well as more recent publications, in which the effect of prenatal alcohol exposure on postnatal alcohol intake was assessed. As explained in those early reviews, increased alcohol intake has been observed after prenatal exposure to different doses of alcohol, ranging from relatively low to high alcohol concentrations. Those effects have been found in studies in which pregnant dams were given alcohol in a liquid diet made available for 24 h, as well as when alcohol was administered intragastrically in controlled amounts, modeling the so-called “binge drinking” of alcohol. In terms of the period of exposure, most of those studies show that exposure to alcohol during the entire gestation period of the rat (22 days), or only during the last 2 weeks, induces high alcohol consumption in the offspring. But even short binge-like exposures to relatively moderate alcohol doses, restricted to the final gestation days (GD 17–20), have systematically resulted in heightened alcohol intake. These results have been observed at different postnatal stages, infancy, adolescence, and adulthood. In general, there are more studies reporting this increased alcohol intake effect when tested early in ontogeny than in adulthood. However, there are some showing that this effect can be directly detected in late adolescence and even in adulthood, although in some cases postnatal re-exposure to alcohol seems necessary. The importance of other factors such as sex, genetic differences, and stress conditions at testing, on the detection of an effect of increased alcohol intake after prenatal alcohol exposure, has also been thoroughly discussed in those reviews (Spear and Molina, 2005; Chotro et al., 2007; Abate et al., 2008).

**Table 1:** Studies in rodents measuring alcohol intake after prenatal alcohol exposure.

Reference	Prenatal period	Alcohol dose	Sex and test age (PD)	Outcome
Bond and Di Giusto (1976)	Whole gestation	Liquid diet with 6.5% (14 g/kg/day)	F56 and 70	Increased alcohol intake
Phillips and Stainbrook (1976)	Whole gestation plus lactation up to weaning	Chablis wine as sole liquid source	F170	Increased Chablis wine intake
Holloway and Tapp (1978)	GD 3 or 15 to PD 24, or to birth	Liquid diet 35% EDC	F-M28 and 70	Increased alcohol intake
Abel and York (1979)	From GD 10 to birth	1–2 g/kg i.g. daily	F150	No increase in alcohol intake
Buckalew (1979)	Whole gestation + lactation up to weaning	5% as sole liquid source	F28	Preference for alcohol over water
Randall et al. (1983)	GD 8–birth	Liquid diet 28% EDC (26–33 g/kg/day)	F-M25	Increased alcohol intake
Nelson et al. (1983)	Whole gestation	Not specified	F-M100	Increased alcohol intake, but under stress
Nash et al. (1984)	Whole gestation	10% as sole liquid source	M90	Increased alcohol intake
McGivern et al. (1984)	GD 7–birth	Liquid diet 35% EDC (14 g/kg/ day)	F-M120	No increase in alcohol intake
Reyes et al. (1985)	Whole gestation	Liquid diet 20.9 EDC (16.85 g/kg/day)	F-M45	No increase in alcohol intake
Grace et al. (1986)	Either weeks 1, 2, 3, or whole gestation	2.8–3.5 g/kg/day	F-Maprox. 120	Increased alcohol intake
Hilakivi (1986)	Whole gestation	7% on weeks 1–2, and 12%, on week 3, as sole liquid source	M64	No increase in alcohol intake
Hilakivi et al. (1987)	Whole gestation	5% on week 1 and 10% on weeks 2–3	M90	Increased alcohol intake in ANA but not in AA rats
Molina et al. (1987)	GD 8	Two i.p. injections, 2.82 g/kg with 4 h–interval	F-M65–75	Increased alcohol intake
Lancaster and Spiegel (1989)	Whole gestation	Beer (50 ml/day or more, 9–11 g/kg/day)	F-M85	Increased beer intake
Molina et al. (1995)	GDs 17–20	1 or 2 g/kg i.g. daily	F-M15	Increased alcohol intake
Domínguez et al. (1998)	GDs 17–20	1 or 2 g/kg i.g. daily	F-M14	Increased alcohol intake
Honey and Galef (2003)	GD 7–birth	4% as sole liquid source	F-M26	Increased alcohol intake, but only if exposed on weaning
Chotro and Arias (2003)	GD 17–20	1 or 2 g/kg i.g. daily	F-M15 and 28	Increased alcohol intake
Arias and Chotro (2005a)	GD 17–20	2 g/kg i.g. daily	F-M14–15	Increased alcohol intake
Arias and Chotro (2005b)	GD 17–20	2 g/kg i.g. daily	F-M14–15	Increased alcohol intake
Pueta et al. (2005)	GD 17–20	2 g/kg i.g. daily	F-M15–16	Increased alcohol intake, but only if exposed on lactation
McMurray et al. (2008)	GD 5–20	Liquid diet 35% EDC + nicotine	F-M30–60	Increased alcohol intake, but only in female rats
Chotro et al. (2009)	GD 17–20	3 g/kg i.g. daily	F-M9–10 or 12–13	Increased alcohol intake
Youngentob and Glendinning (2009)	GD 11–20	Liquid diet 35% EDC	F-M30 and 90	Increased alcohol intake
Díaz-Cenzano and Chotro (2010)	GD 17–18 or GD 19–20	2 g/kg i.g. daily	F-M14 and 26–27	Increased alcohol intake in subjects exposed on GD 19–20
Shea et al. (2012)	Pre–pregnancy Whole gestation	5% + 1 g/l sucralose as sole liquid source	F-M40–45 to 70	Increased alcohol intake
Youngentob et al. (2012)	GD 6–10 or GD 11–20	6.7% as sole liquid source	F-M12–14	Increased alcohol intake
Abate et al. (2014)	GD 17–20	2 g/kg i.g. daily	F-M14–15	Increased alcohol intake, particularly in females
Díaz-Cenzano et al. (2014)	GD 19–20	2 g/kg i.g. daily	F-M14	Increased alcohol intake
Nizhnikov et al. (2014)	GD 17–20	1 g/kg i.g. daily	F-M14–15	Increased alcohol intake
Miranda-Morales et al. (2014)	GD 17–20	1 g/kg i.g. daily	F-M5	Increased alcohol intake
Fabio et al. (2015)	GD 17–20	2 g/kg i.g. daily	F-M37–62	Increased alcohol intake
Nizhnikov et al. (2016)	GD 17–20	1 g/kg i.g. daily	F-M14 (F1, F2 and F3)	Increased alcohol intake on all generations
Gaztañaga et al. (2017)	GD 17–20	2 g/kg i.g. daily	F-M14	Increased alcohol intake
Biggio et al. (2018)	GD 17–20	1 g/kg i.g. daily	M30–85 or 90–145	No increase in alcohol intake, but alcohol preference after maternal separation
Fernández et al. (2019)	Whole gestation + postnatal week 1	10% sole liquid source 22 h/day + water 2 h/day	M56–84	Increased alcohol intake from first testing trials, and potentiates isolation effects on alcohol intake
Gore-Langton and Spear (2019)	GD 17–20	2 g/kg i.g. daily	F-M35 and 56–60	Increased alcohol intake in adolescent and adult males
Wille-Bille et al. (2020)	GD 17–20	2 g/kg i.g. daily	F-M30–50	Increased alcohol intake and preference, but only males reared in enriched environment

*GD, gestational day; PD, postnatal day; i.g., intragastric; F, females; M, males*.

During the last decade, new studies showing evidence of an increased intake of alcohol following prenatal alcohol exposure in rodents have been added to those reviewed previously. Among all of this literature, those studies proposing and testing possible mechanisms by which fetal exposure to alcohol may increase the avidity for this drug are of particular interest for this review.

## Possible Mechanisms

On the basis of the results found in animal studies, a number of different mechanisms have been proposed to explain the increased consumption of alcohol that is observed following prenatal alcohol exposure. Some propose an indirect link between high alcohol consumption and other alterations induced by prenatal alcohol exposure, while others suggest more direct pathways between prenatal alcohol consumption and an increased acceptance of the drug.

### Indirect Mechanisms

Genetic differences may lead to differences in susceptibility to the teratogenic effects of alcohol. For example, in a study using two lines of rats selected to differ in alcohol intake (AA, alcohol-preferring, and ANA, alcohol avoiding rats) prenatal alcohol exposure was found to differentially affect voluntary alcohol consumption (Hilakivi et al., 1987). This difference was explained by differences in alcohol metabolism between both ratlines. However, the authors did not offer any explanation for the connection between this differential susceptibility to the teratogenic effects of alcohol and the increased alcohol consumption. Epigenetic alterations have also been proposed as a mechanism by which prenatal alcohol exposure results in increased alcohol intake. The transgenerational transmission of the effects induced by prenatal alcohol exposure was investigated in a study including three generations of rats (Nizhnikov et al., 2016). Pregnant rats were administered with 1 g/kg of alcohol from GD 17–20, and the offspring of the first generation (F1) was tested in terms of alcohol intake and sensitivity to alcohol-induced sedation in comparison with water-exposed or untreated subjects. These F1 subjects were mated and their descendants (F2) were tested on those same measures and subsequently used to produce F3, which was also tested. The results of these tests revealed that alcohol intake increased in all three generations and that the effects on sedation were observed in F1 and F2, but not in F3. On the basis of previous research (Popoola et al., 2015), differences in maternal care were ruled out as a cause of this transgenerational transmission of the altered response to alcohol. However, in the discussion of the results, no alternative mechanisms were proposed to explain the interesting results observed in the F2 and F3 subjects. Transcript and epigenetic changes as a consequence of prenatal alcohol exposure have been described in several studies with animals (for a complete review, see Comasco et al., 2018), and these epigenetic modifications could underlie the results of the study conducted by Nizhnikov et al. (2016). Exploration of the mechanisms linking these genomic alterations and the increase in alcohol intake in subjects prenatally exposed to alcohol constitutes an interesting research topic that requires rigorous investigation. Recently, preliminary steps have been taken to clarify this issue with a study analyzing the protective effects of environmental enrichment upon modulation of gene expression, the anxiety response, and alcohol intake produced by prenatal alcohol exposure (Wille-Bille et al., 2020). The results showed that prenatal alcohol-induced upregulation in the kappa opioid receptor system mRNA levels in the amygdala, as well as prodynorphin mRNA levels in the ventral tegmental area, with the latter effect being linked to lower DNA methylation at the gene promoter. These effects were normalized by postnatal environmental enrichment manipulations. In addition, environmental enrichment also had a protective effect on alcohol intake, this effect being more marked in males than in females.

Prenatal alcohol has been found to alter the normal development of the neurochemical systems. Based on data showing that voluntary alcohol intake is partially regulated by the activity of the monoaminergic system (Ericson et al., 1998; Gonzales and Weiss, 1998) and that the acquisition of alcohol consumption habits is mediated by the dopaminergic mesencephalic system (Gianoulakis, 2001), it has been proposed that the altered alcohol intake after prenatal exposure is due to alterations in this system. Prenatal alcohol exposure has been observed to affect the development of the dopaminergic system (Shen et al., 1999; Aghaie et al., 2019), inducing, for example, hyperactivity (Cheng et al., 2018) together with sensitivity to the stimulant effects of alcohol (Becker et al., 1993; Barbier et al., 2009). These effects, together with other physiological and behavioral effects, have been linked to increased alcohol consumption in subjects prenatally exposed to alcohol.

Other neurochemical systems that have been shown to be related to motivational aspects of alcohol consumption, either directly or indirectly, and that are affected by alcohol exposure are GABA, serotonin, and the opioid system (Alfonso-Loeches and Guerri, 2011). Specifically, it has been demonstrated that the opioid system is involved in the increased alcohol intake observed in subjects exposed prenatally to a relatively low alcohol dose (1 g/kg; Nizhnikov et al., 2014). In this study, it was observed that prenatal alcohol exposure, in addition to increased alcohol intake in the offspring, induced changes in the kappa opioid receptor system. In particular, a decrease in synaptosomal kappa-opioid receptor expression was found in brain areas implicated in the response to alcohol. The authors suggest that these changes in kappa-opioid function and expression are involved in the enhanced postnatal alcohol intake observed following prenatal exposure.

Differences in the response to stress have been proposed as an alternative way to explain the increased consumption of alcohol following prenatal exposure. Some studies have linked stress and alcohol consumption *via* alterations in the development of the HPA-axis and the pituitary ß-endorphin system (Prasad and Prasad, 1995; Fahlke et al., 2000; Nash and Maickel, 2013). For instance, in one study, prenatal alcohol exposure was found to induce higher alcohol intake, but only when rats were tested after chronic stress (Nelson et al., 1983). Interestingly, in most studies, alcohol intake is tested in conditions of isolation, a stressful situation for rats that could facilitate the observation of differential effects of alcohol consumption. Prolonged isolation housing during early development has also been shown to induce augmented alcohol intake in rodents (Lopez et al., 2011; Kutcher et al., 2016). Additionally, in one study it was found that this heightened alcohol intake was facilitated in adolescent males exposed to alcohol during gestation and the first week of lactation (Fernández et al., 2019). In that case, males pre- and postnatally exposed to alcohol showed significantly higher alcohol intake and increased alcohol preference in comparison with non-exposed controls during the first 5–6 testing sessions, but only if they had been housed in isolation conditions from weaning.

The anxiolytic effects of alcohol have also been considered as an explanation for the high alcohol consumption observed during stressful situations, particularly in adolescence (Spear and Molina, 2005). Recently, it has been found that adolescent rats exposed prenatally to alcohol were more sensitive to the social facilitation and anxiolytic effects of acute alcohol (Mooney and Varlinskaya, 2018). Another study examined the combined effects of prenatal alcohol exposure and postnatal maternal separation on HPA responsiveness, anxiety behavior, and alcohol intake, in male offspring (Biggio et al., 2018). The results of this study revealed that male subjects exposed prenatally to alcohol (1 g/kg) did not display an increased intake or preference for alcohol in comparison with non-exposed subjects or those that experienced only maternal separation. Maternal separation by itself was found to increase intake of low concentrations of alcohol, whereas adult males subjected to both treatments —prenatal alcohol and maternal separation—displayed an increase in anxiety-related behavior and an increased preference for alcohol at either low or high concentrations. The failure to observe an effect on alcohol intake in males following prenatal alcohol exposure is in accordance with the findings of another study in which a sex-dependent result was found after alcohol prenatal exposure, as a function of alcohol dose and age of testing (Chotro and Arias, 2003). This study found that whilst at infancy both males and females exposed prenatally to either 1 or 2 g/kg alcohol showed increased alcohol intake, at adolescence, only males exposed to the higher dose and females exposed to the 1 g/kg dose drank more alcohol than non-exposed subjects. It is possible that an increase in the effect of alcohol consumption might have been observed in the study by Biggio et al. (2018) if the female siblings had been included in the test.

### Learning Mechanisms

Other mechanisms proposed to account for the heightened alcohol intake observed after prenatal exposure are based on the notion that the fetus may learn about different aspects of alcohol during exposure in the amniotic environment and this may have an impact on the postnatal response to the drug. Both human and animal fetuses detect chemosensory stimuli that enter the amniotic fluid from the maternal diet, and it has been shown that this experience may change the subsequent response to those flavors (Faas et al., 2000; Schaal et al., 2000; Mennella et al., 2001). Rat fetuses in the final days of gestation (GD 17 to birth) can acquire and express basic forms of non-associative and associative learning (Smotherman, 1982; Smotherman, 2002a,b; Stickrod et al., 1982; Smotherman and Robinson, 1985, 1988a,b; Gruest et al., 2004). Alcohol is one of those substances with chemosensory properties that is able to cross the placenta with ease and reaches not only the fetal tissues but also accumulates in the amniotic fluid, from where it is slowly eliminated (Chotro et al., 2009; Burd et al., 2012). Therefore, after maternal alcohol ingestion, the fetus is exposed to the flavor of alcohol as well as its pharmacological effects. Some studies explain postnatal increased alcohol intake in terms of a mere stimulus exposure effect, that is, familiarity with the alcohol flavor, or habituation to neophobia, which facilitates the initial acceptance of the particular chemosensory aspects of alcohol (Spear and Molina, 2005; Díaz-Cenzano and Chotro, 2010). However, this mechanism alone is not sufficient to explain the increased alcohol consumption observed when subjects are tested repeatedly and/or after a long period after the prenatal experience (Fabio et al., 2015; Gaztañaga et al., 2015).

#### Associative Learning Mechanisms

A mechanism that has been broadly investigated and has obtained abundant support in the last two decades, is one that links increased alcohol consumption to fetal associative learning about alcohol (Chotro and Arias, 2003; Arias and Chotro, 2005a,b, 2006; Chotro et al., 2007, 2009; Díaz-Cenzano and Chotro, 2010; Miranda-Morales et al., 2010; Youngentob et al., 2012; Díaz-Cenzano et al., 2014; Bordner and Deak, 2015; Gaztañaga et al., 2015). The working hypothesis of most of these studies begins with the assumption that the fetus acquires an appetitive conditioned response to alcohol by the formation of an association between the flavor of alcohol (the conditioned stimulus) and its pharmacological effects (the reinforcer).

##### The Role of the Opioid System

With regard to the pharmacological effects of alcohol, several studies have explored the implied role of the endogenous opioid system. This neurochemical system is known to play an important role in alcohol consumption behaviors (Gianoulakis, 2001, 2004) and in the mediation of the reinforcing effects of alcohol, particularly the mu-opioid receptor system (Acquas et al., 1993; Stromberg et al., 1998; Gianoulakis, 2001; Molina-Martínez and Juárez, 2020). Based on this body of evidence, the reinforcing properties of alcohol during gestation were tested by manipulating the prenatal opioid system. The results of several studies have shown that blocking the opioid receptor system with a non-selective antagonist (naloxone or naltrexone) during prenatal alcohol exposure prevented the observation of the increased alcohol intake effect in the offspring (for example, Chotro and Arias, 2003; Youngentob et al., 2012).

The fetal opioid system has also been found to be activated by the amniotic fluid, in the absence of alcohol. It has been proposed that the amniotic fluid contains a substance that stimulates the kappa-opioid receptors, known as KIF (kappa inducing factor), which is functional in the last two gestational days (GD 20–21; Méndez-Gallardo and Robinson, 2010). These researchers suggested that KIF could be the agent that mediates the preferences acquired by flavors experienced prenatally in the amniotic fluid, including the enhanced acceptance of alcohol observed after prenatal exposure to this drug. This hypothesis was tested in further studies in our laboratory. Taking into account that activity of KIF has been reported to start at around GD 20, the prenatal administration of alcohol prior to these days (GDs 17–18) would not be expected to produce the effect of increased intake, whereas this would be observed when alcohol exposure occurs on the following days (GDs 19–20). The results appear to support the hypothesis that KIF, and therefore, the stimulation of kappa-opioid receptors, could play an important role as the reinforcer in this prenatal learning (Díaz-Cenzano and Chotro, 2010). In a second study, this hypothesis was tested from a more unambiguous perspective. Considering that KIF acts directly on kappa-opioid receptors, while the reinforcing effects of alcohol are mediated by the stimulation of mu-opioid receptors, the reinforcing effects of the amniotic fluid (with KIF) and alcohol were assessed by using specific antagonists for each receptor system. The results demonstrated that when prenatally blocking mu-opioid receptors during alcohol exposure, the usually observed effect of increased postnatal consumption of alcohol was completely abolished. However, the blockage of the kappa-opioid receptor system did not abolish this effect. These results allow us to rule out the possibility that the proposed effects of the amniotic fluid (and KIF) on the opioid system is the positive reinforcer responsible for the appetitive conditioned response. The pharmacological effects of alcohol on the mu-opioid receptor system were instead found to be critical for observing the increased consumption of alcohol after prenatal exposure (Díaz-Cenzano et al., 2014). Coincidently, an increase in the activity of mu-opioid receptors in the ventral tegmental area was reported after prenatal alcohol exposure, together with augmented alcohol intake in adolescence (Fabio et al., 2015).

##### Acetaldehyde as the Reinforcer

Once this was confirmed, further research aimed to clarify whether the reinforcer responsible for the activation of the opioid system is either alcohol itself or its first metabolite acetaldehyde. A growing body of research indicates that many of the deleterious effects of alcohol on gestation are actually produced by acetaldehyde (Sreenathan et al., 1982; Webster et al., 1983; Eriksson, 2001). As mentioned previously, during gestation alcohol freely crosses the placenta, reaching all fetal tissues to the same extent as maternal blood, including the brain (Zorzano and Herrera, 1989). From there, alcohol is eliminated, mostly unchanged, through maternal metabolism (Clarke et al., 1986). Fetal alcohol hepatic capacity is minimal or null, and therefore peripheral acetaldehyde is not produced by the fetus, while acetaldehyde produced by the maternal liver does not cross the placenta (Heller and Burd, 2014). However, in the fetal brain, acetaldehyde is produced in abundance from alcohol, mainly by the catalase system (Hamby-Mason et al., 1997). Several studies have demonstrated the important role of acetaldehyde in the pharmacological and behavioral effects of alcohol. It has also been found that acetaldehyde produced in the peripheral circulation (in the liver) and centrally (in the brain) have distinct and opposing behavioral effects: peripheral acetaldehyde induces aversive effects (Quertemont and Tambour, 2004) whereas central acetaldehyde is involved in the reinforcing appetitive properties of alcohol (for a review, see Hahn et al., 2006; Correa et al., 2012). Hence, the balance between peripheral and central acetaldehyde derived from alcohol consumption may be critical in determining the perceived effect of alcohol intoxication and may influence further intake of this drug.

Based on all of these facts related to alcohol metabolism, some studies have assessed the role of acetaldehyde on the increased alcohol consumption observed following its prenatal administration. In the infant and newborn rat, it has been found that acetaldehyde produced from alcohol by catalases in the brain is responsible for the reinforcing effects of alcohol (Nizhnikov et al., 2007; March et al., 2013a,b). The participation of centrally produced acetaldehyde has also been investigated in the prenatal period by administering to the pregnant rat the acetaldehyde sequestering agent D-Penicillamine together with alcohol. The results show that in the absence of acetaldehyde prenatal alcohol, exposure does not induce an increase in postnatal alcohol consumption. These results confirmed that acetaldehyde, and not alcohol, is the main reinforcer and that its production is critical for the occurrence of prenatal appetitive learning about alcohol (Gaztañaga et al., 2017; Chotro et al., 2019). Considering the outcomes of these studies, it could be hypothesized that the reinforcing properties of prenatal alcohol are produced by central acetaldehyde, which in turn stimulates the endogenous opioid system. This hypothesis is supported by studies showing that the reinforcing effects of acetaldehyde produced in the brain from alcohol may be mediated by the μ-opioid receptor system, and acetaldehyde has been found to stimulate the release of ß-endorphins (Font et al., 2013; Xie et al., 2013). In addition, the condensation product of acetaldehyde and dopamine, salsolinol (Ito et al., 2018), has been found in the fetal brain following chronic prenatal alcohol exposure (Mao et al., 2013). Salsolinol has been shown to be involved in the motivational effects of alcohol and its high intake and produces its effect by interacting with the μ-opioid receptors in the posterior ventral tegmental area (Xie et al., 2012; Quintanilla et al., 2014; Peana et al., 2017), Therefore, the monoamine system appears to be directly implicated in the reinforcing effects of alcohol, and, consequently in prenatal learning about alcohol; although the role of this system has not yet been fully investigated. This could yet prove to be the missing link between the reinforcing action of acetaldehyde and the activation of the opioid system.

The appetitive learning acquired *in utero* after alcohol exposure can also account for the increased preference or enhanced behavioral response to the odor of alcohol observed from newborns to adult rats (Youngentob et al., 2007; Eade et al., 2009, 2010; Middleton et al., 2009; March et al., 2013b; Gaztañaga et al., 2015). Interestingly, similar results have been reported in humans. For example, the newborns of mothers who frequently consumed alcohol during gestation responded to alcohol odor with more appetitive facial reactions than babies from control mothers who were infrequent consumers (Faas et al., 2000, 2015). In another study, it was found that young adults with prenatal history of alcohol exposure rated alcohol odor as more pleasant than non-exposed control subjects (Hannigan et al., 2015).

In addition to the enhanced response to alcohol odor, the prenatal experience has been observed to increase the reinforcing capacity of alcohol in operant conditioning tasks (March et al., 2009; Miranda-Morales et al., 2010; Gaztañaga et al., 2015); and has also been shown to interact with postnatal conditioning, potentiating appetitive learning about alcohol and retarding the acquisition of an aversion to this substance (Arias and Chotro, 2006; Chotro et al., 2009). The enhanced appetitive reinforcing properties of alcohol, together with the development of tolerance and reduced sensitivity to alcohol’s aversive effects, have also been proposed as mechanisms by which prenatal alcohol may lead to high postnatal alcohol consumption (Arias et al., 2008; Pautassi et al., 2012; Fabio et al., 2015; Gore-Langton and Spear, 2019).

These mechanisms are not mutually exclusive and the consistent outcomes of all the studies cited here suggest that they could be acting simultaneously to generate the augmented alcohol intake response systematically observed after exposure to alcohol during gestation. The increased alcohol intake described in many studies with human subjects exposed prenatally to alcohol may be partially explained by these same mechanisms that have been experimentally studied with rodents. For instance, those studies in which alcohol odor elicited positive reactions in subjects with prenatal alcohol exposure seem to support the idea of an appetitive response to alcohol acquired before birth (Faas et al., 2000, 2015; Hannigan et al., 2015).

## Concluding Comments and Future Directions

After reviewing all of the evidence from controlled experimental studies with animals, the connection between prenatal alcohol exposure and augmented alcohol intake during infancy, adolescence, and even adulthood appears to be clear. This link suggests that prenatal alcohol exposure increases the probability of early onset of alcohol use, which in turn has been described as a strong predictor of alcohol dependence (Grant, 1998). Although the causal relationship between adolescent-onset and adult alcohol use is still under debate (Prescott and Kendler, 1999), it is clear that adolescence is a vulnerable period for the neurobehavioral effects of alcohol (Spear, 2000, 2015). Unlike what occurs in adult subjects, initiation of alcohol use in adolescence has been found to accelerate the course of alcohol dependence, without the need for a long history of alcohol consumption (Clark et al., 1998; Spear, 2002). Therefore, any situation that favors the initiation of alcohol use during this period of development, such as stress or prenatal alcohol exposure, can be considered a risk factor for later alcohol use and misuse.

Thus, understanding the mechanisms by which prenatal alcohol exposure favors the early initiation of alcohol consumption may allow us to find strategies to mitigate the behavioral effects of this fetal experience. Moreover, it would be interesting to consider prenatal alcohol exposure as a case of involuntary early onset of alcohol use when designing prevention policies. This is particularly important if we assume that (as indicated by the longitudinal studies reviewed here), the sooner that alcohol intake begins, the greater the possibility of a long history of alcohol consumption and hence, the higher the likelihood of developing an alcohol use disorder.

Given these considerations, it is clear that more clinical and preclinical research is needed to explore prenatal alcohol exposure as a causal pathway leading to alcohol disorders in adolescence and adulthood. As discussed in this review, it has been well established that prenatal learning about the sensory and pharmacological properties of alcohol is a mechanism that plays an important role in facilitating the early onset of alcohol consumption. The opioid system has been found to mediate this prenatal learning, in which acetaldehyde acts as the main appetitive reinforcer. Future research should aim to find the link between acetaldehyde and the activation of the opioid system, with the dopaminergic system and/or salsolinol being the main candidates for this role. In addition to fetal alcohol learning, other identified coexistent and interacting mechanisms undoubtedly need to be investigated in more depth. For instance, stress is a factor that interacts with prenatal exposure to alcohol, facilitating in many cases the observed increase in alcohol intake in the exposed offspring. Further research is also needed to identify the causal processes by which prenatal alcohol-induced alterations in the activity of the HPA-axis drive the subject to consume more alcohol. Furthermore, it would be interesting to continue elucidating the role of the anxiolytic effects of alcohol on the increased intake response observed in subjects prenatally exposed to alcohol. Finally, the mechanistic connection between epigenetic modifications induced by prenatal alcohol exposure and the resulting changes in alcohol intake remains an underexplored but promising field of research, which needs to be addressed through rigorous research. In this regard, the first steps have already been taken, as shown, for example, in the results of a study already mentioned in this review (Wille-Bille et al., 2020).

## Author Contributions

MG, AA-A, and MC contributed equally to manuscript writing, revision, read and approved of the submitted version.

## Conflict of Interest

The authors declare that the research was conducted in the absence of any commercial or financial relationships that could be construed as a potential conflict of interest.
